# Effect of Different Implant Designs on Strain and Stress Distribution under Non-Axial Loading: A Three-Dimensional Finite Element Analysis

**DOI:** 10.3390/ijerph17134738

**Published:** 2020-07-01

**Authors:** Hélder Oliveira, Aritza Brizuela Velasco, José-Vicente Ríos-Santos, Fernando Sánchez Lasheras, Bernardo Ferreira Lemos, Francisco Javier Gil, Alexandrine Carvalho, Mariano Herrero-Climent

**Affiliations:** 1 Faculty of Health Sciences, University Fernando Pessoa, 4200-150 Porto, Portugal; helderol@ufp.edu.pt (H.O.); blemos@ufp.edu.pt (B.F.L.); alexcarv@ufp.edu.pt (A.C.); 2 Department of Surgery and Medical-surgical Specialties, University of Oviedo, 33006 Oviedo, Spain; aritzabrizuela@hotmail.com; 3 Department of Periodontology, University of Seville, School of Dentistry, 41009 Sevilla, Spain; 4 Mathematics Department, Faculty of Sciences, University of Oviedo, Oviedo 33007, Spain; sanchezfernando@uniovi.es; 5 Bioengineering Institute of Technology, International University of Catalonia, 08017 Barcelona, Spain; xavier.gil@uic.cat; 6 Porto Dental Institute, 4150-518 Oporto, Portugal; dr.herrero@herrerocliment.com

**Keywords:** dental implants, design, finite element analysis, strain distribution, stress distribution, bone quality

## Abstract

Implant design evolved alongside the development of implant therapy. The purpose of this finite element analysis (FEA) study was to analyze the influence of different implant designs on the stress and strain distribution to the implants and surrounding bone. Three implant designs with the same length and diameter were used. The three-dimensional geometry of the bone was simulated with a cortical bone of three different thicknesses and two medullar bone densities: low density (150 Hounsfield units) and high density (850 Hounsfield units). A 30° oblique load of 150 N was applied to the implant restoration. Displacement and stress (von Mises) results were obtained for bone and dental implants. The strain and stress distributions to the bone were higher for the tissue-level implant for all types of bone. The maximum principal strain and stress decreased with an increase in cortical bone thickness for both cancellous bone densities. The distribution of the load was concentrated at the coronal portion of the bone and implants. All implants showed a good distribution of forces for non-axial loads, with higher forces concentrated at the crestal region of the bone–implant interface. Decrease in medullar bone density negatively affects the strain and stress produced by the implants.

## 1. Introduction

Dental implants are devices widely used in routine dental practice. Their high success rate in different indications and protocols (89–100%) has facilitated their incorporation into the treatment plans of patients who need replacement of missing teeth. Oral implantology must satisfy the needs of patients and clinicians regarding functional, comfort, and aesthetic aspects [1,2,3,4].

After the implants are inserted into the bone, an interaction occurs between the surface of the implant and the bone that surrounds it, ending in what is known as osseointegration. Osseointegration is defined as the direct and structural connection between living and structured bone and the surface of an implant subjected to a functional load [5]. Achieving osseointegration requires several requirements: biocompatible material, adequate macrodesign, appropriate surface, correct surgical technique, and adequate implant loading [6,7]. Important aspects of the biocompatibility of the materials were highlighted a recent publication [8].

Osseointegration will be conditioned by biomechanical stimuli that will directly affect bone–implant contact. The areas of the implant that come into contact with the surrounding bone will be responsible for the distribution of loads. The design of dental implants has two purposes: to promote the correct primary stabilization, since osseointegration is based on having adequate mechanical stability and a favorable biological environment, and to favor the correct transfer of loads to the tissues surrounding the implant, once secondary or biological stability has been achieved after osseointegration [9,10].

The macrodesign includes several parts of the implant such as the morphology of the implant, the nucleus or body, the shape and depth of the threads, and the thread pitch, among others. The shape and geometry of one of these components will influence both the distribution of loads to the bone surrounding the implant and the generation of stresses at different levels of the implant. Tapered implant bodies and triangular compacting threads are factors in implant design related to a higher primary stability [11].

In current implantology, the use of what is known as internal connection is widespread. Different elements are connected to the body of the implant inside, therefore requiring a hollow bone with a practicable thread, which allows accommodation of the different abutments or elements that facilitate prosthesis construction. Within what is known as internal connection implants, we find two types of conceptually different implants: 1—tissue-level implants or implants in one piece, which incorporate the transepithelial portion to the body of the implant without continuity solution; 2—bone-level implants, or implants with a switching platform concept, in which the transepithelial portion is in a different element (abutment) that is incorporated into the body of the implant, this being slightly narrower than the more coronal portion of the implant [12,13,14]. In such a way, these designs will show different biomechanical behaviors to the supported forces: on the one hand, in terms of the distribution of loads to the bone that surrounds it and, on the other, in terms of the stresses suffered by the implant itself.

Clinical practice has routinely incorporated treatment protocols that require specific conditions, such as post-extraction implants and the application of immediate loading protocols. In both cases, it is necessary to achieve high implant stability values after insertion (primary stability), which avoid micromovements greater than 150 µ [15,16,17,18].

The influence of the implant macrodesign will be decisive in achieving adequate primary stability. The application of immediate loading protocols entails the transmission of efforts from the first moment after insertion of the implants, as well as the generation of stresses to the implant itself [19,20,21,22]. Several authors have assessed the impact of implant macrodesign, and the different factors that influence it, on the primary stability of implants and, therefore, on the achievement of osseointegration [21,23].

The characteristics of the bone surrounding the implants will be another essential factor to consider in primary stability. At the level of the bone that will receive the implants, there are two areas with different characteristics: the cortical bone and the cancellous or medullar bone. Primary stability will, therefore, depend on the macrodesign of the implant, the characteristics of the bone that houses it, and the loads on the implant and how they are distributed.

Greater tapering of the implant body will facilitate greater implant stability, as well as a more favorable insertion, facilitating the surgical protocol [24,25]. In some specific clinical situations where greater implant stability is required (supposedly more unfavorable conditions), tapered implants show some advantages. These situations could include application of immediate loading protocols, post-extraction implants, placement of implants in low-density bone, and so forth [26,27]. It is very difficult to clinically detect the distribution of stress occurring on the implant and bone as a result of the force on dental implants. Therefore, the use of numerical methods, such as finite element analysis (FEA) makes it possible to evaluate the stress on dental implants and surrounding bone.

In the present study, a three-dimensional (3-D) FEA was used, in order to analyze the stress and strain occurring on the dental implants and surrounding bone under non-axial load (most unfavorable situation) [28,29,30]. According to Chung et al., the maximum effective stress induced by an oblique load could be twice as high as the maximum effective stress caused by an equal amount of vertical load [31].

The purpose of this FEA study was to analyze the influence of different implant designs on the stress and strain distribution to the implants and surrounding bone.

## 2. Materials and Methods

A finite element assessment requires definition of the parameters that characterize the model in which the study is carried out.

The three-dimensional (3D) geometry of the bone was simulated with cortical bone of three different thicknesses—0.5 mm, 1 mm, and 2.0 mm—and medullar bone with a density at two levels—low density (150 Hounsfield units—HU) and high density (850 Hounsfield units—HU).

**Figure 1:**
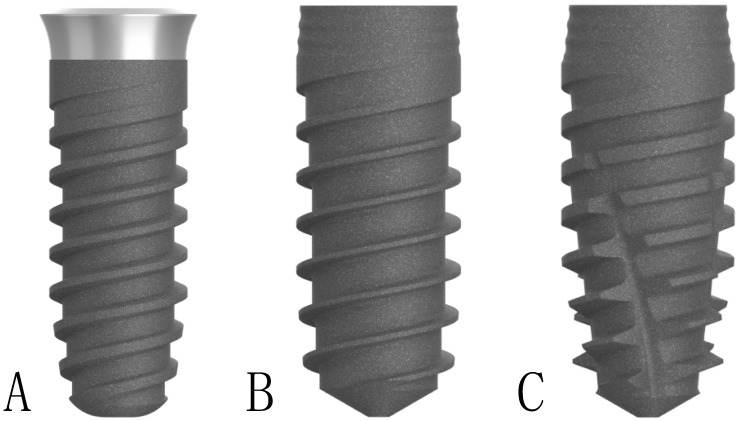
Implant designs evaluated in the study: Essential Cone^®^ (**A**), Vega^®^ (**B**), and Vega+^®^ (**C**).

The implants evaluated in this study were for daily use in dental practice, the Klockner Implant System^®^ (SOADCO, Escaldes-Engordany, Andorra), with the corresponding CE marking (Figure 1).

Essential Cone^®^ (from now on designated as implant A) with a 1.5 mm neck or polished portion was used. It is a one-piece tissue-level concept implant, which incorporates the transepithelial portion attached to the implant body without continuity solution, eliminating the gap or implant-abutment connection. This is an internal connection implant system made of commercially pure grade III titanium. It has a tapered implant apex, straight body, double pitch thread (2.2 mm), 45° trapezoidal thread profile, tapered thread core at the apex, is straight in the middle of the body, and is conical in the most coronal part. The neck of the implant is concave in the shape of a tulip and has a micro-thread (1.5 mm), with a pitch of 0.05 mm.

Vega^®^ (from now on designated as implant B) is a two-piece bone-level implant concept that applies the switching platform treatment philosophy, seeking better maintenance of the crestal bone level after insertion. This implant is made of a new generation of grade IV titanium, known as OPTIMUM^®^ titanium, and is a cold hardening type. The conical design of the implant, in its most coronal portion, allows a better distribution of loads to the adjacent bone tissue. The micro-grooves dissipate stress in the crestal portion, preventing bone loss when implant loading occurs, thus helping to maintain the bone level. It has a tapered implant apex, straight body, double pitch thread (2.2 mm), 24° trapezoidal thread profile, tapered thread core at the apex, is straight in the middle of the body, and is conical in the most coronal part. The implant neck is convex cone-shaped and has three rings (0.3 mm) with a 0.4 mm gap between them.

Vega+^®^ (from now on designated as implant C) is also a bone-level concept implant in two pieces that applies the switching platform treatment philosophy, like implant B. Its design tries to facilitate insertion at the bone site, as well as to achieve greater primary stability. It is an implant that seeks to obtain greater primary stability, thanks to a series of changes in its design: more conical nucleus, 0.2 mm greater diameter in its coronal third, threads of greater width, vertical grooves that allow bone compaction when the implant is inserted, and is a more self-tapping implant. Implant C is also made of OPTIMUM^®^ titanium. It has a tapered implant apex, straight body, double pitch thread (2.2 mm), 34° trapezoidal thread profile, tapered thread core at the apex, is straight in the middle of the body, and is conical in the most coronal part. The implant neck is convex cone-shaped and has three rings (0.3 mm), with a 0.4 mm gap between them.

The size of the implants used were 4 mm in diameter and 10 mm in length for all types of implants.

On them, the connection of an abutment for elaboration of a 6 mm height cemented prosthesis was considered, according to the design suggested and commercialized by the manufacturer. A crown was placed on the aforementioned abutment that reproduced a first upper premolar with an inclination of its cusps of 6°. The cement layer between the crown and abutment was ignored, assuming a precise passive fit and an effective joining of the two components.

The implants were placed at the recommended levels with respect to the bone crest.

The loading protocol simulated an intimately connected bone–implant interface (osseointegration simulation, delayed loading protocol).

The material properties were assumed to be homogeneous, isotropic, and linearly elastic, and the applied state equation was Hooke Law through Young and Poisson Modulus specification (Table 1). An adaptative mesh method was used to define the mesh model, with a maximum element size of 1 mm for the medullar bone constraint zones, 0.5 mm for models with 1.00 mm thickness in the cortical bone area, and 0.25 for those cortical bones belonging to models with only 0.5 mm thickness area. This ensures at least three nodes were used as information points in each line path. About 290 000 nodes were created for each model using the FEA software program (Ansys 2020 R1; Ansys Inc.).

**Figure 2:**
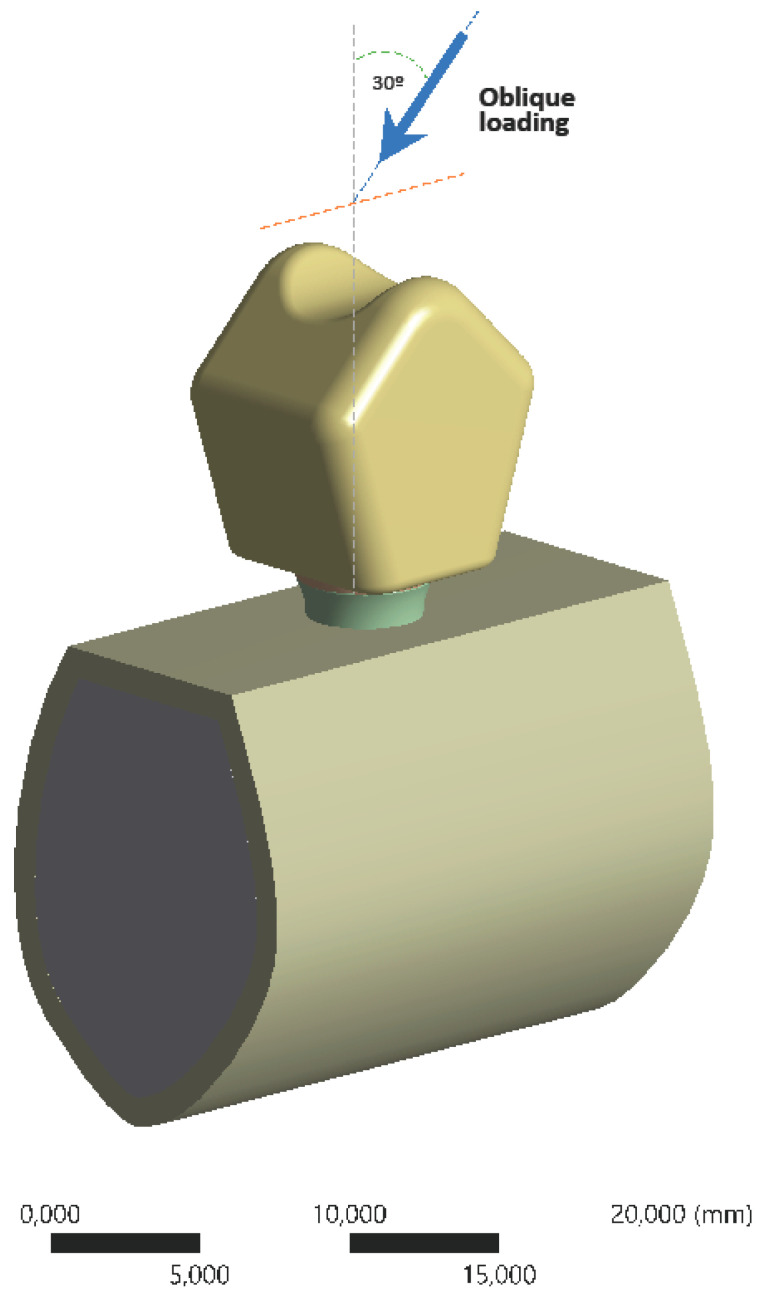
Boundary and load conditions of the FE model. Total force applied of 150 N. FE, finite element.

Displacement and stress (von Mises) results were obtained for bone and dental implants. The force between the alveolar crest and the implant was pushed downside, with a 30° oblique load of 150 N, avoiding stress concentration errors, and the sides of the alveolar bone were used as a fixed support constraint (Figure 2). The load was applied at the level of the central fossa of the restoration, and the model was fixed in the support bone.

Convergence displacement–stress U–S criteria with Newton Raphson residual control were used. Most models attained less than 0.2% convergence criteria, meaning the convergence criterion was set to less than 0.2% in the changes in the total deformation energy of all the elements. Shape control and mesh quality were measured and refined, reaching a final aspect ratio between 0.75 and 0.88. A convergence test with the finite element models was carried out to verify the quality of the mesh. The convergence criterion was set to less than 1% in the changes in the total deformation energy of all the elements. Based on the results of the convergence test, an average element size of 0.6 mm was established for the mesh in all finite element models.

## 3. Results

### 3.1. Strain Distribution in Bone

**Figure 3:**
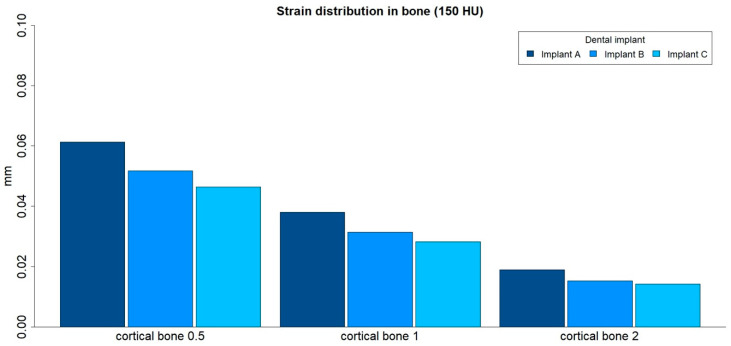
Strain distribution (mm) in low-density medullar bone (150 HU) for all cortical thicknesses (0.5 mm, 1 mm, and 2 mm) in all implant designs: A, B, and C.

**Figure 4:**
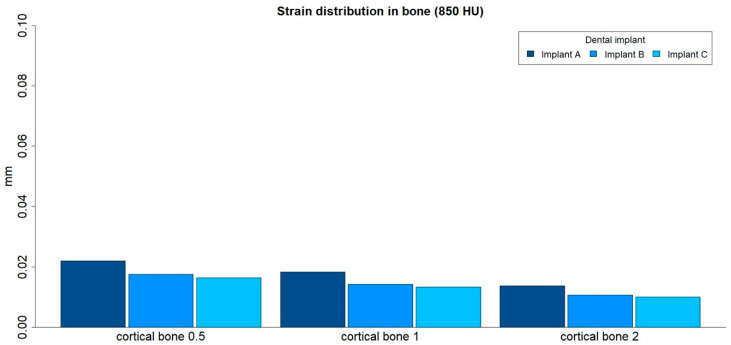
Strain distribution (mm) in high-density medullar bone (850 HU) for all cortical thicknesses (0.5 mm, 1 mm, and 2 mm) in all implant designs: A, B, and C.

The strain distributions in the bone were analyzed in both medullar bone densities (150 HU and 850 HU), for all cortical bone thicknesses (0.5 mm, 1 mm, and 2 mm), and for all implant designs (Figure 3 and Figure 4; Table 2).

The strain distribution to the bone was higher for implant A followed by implant B and implant C, respectively, and for all types of bone. Furthermore, the maximum principal strain decreased with an increase in cortical bone thickness for both cancellous bone densities, and this difference was more prominent in the low-density medullar bone.

**Figure 5:**
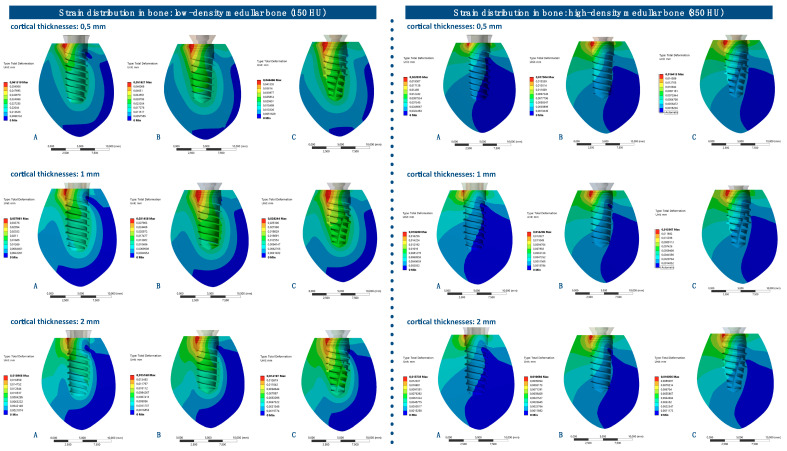
Strain distribution in bone for all implant designs: A, B, and C. Note: the figure is in very high resolution to be able to be enlarged: enlarge it in your word processor or view it in HTML format.

The distribution of the strain to the bone was concentrated, in all the examples, at the level of the most coronal portion of the implants. Likewise, there was a more homogeneous distribution of the strain throughout the body of implant C (Figure 5).

### 3.2. Strain Distribution in Implants

**Figure 6:**
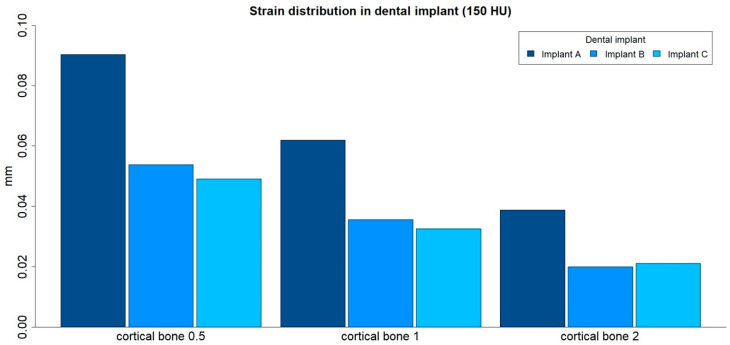
Strain distribution (mm) in implants, in low-density medullar bone (150 HU), for all cortical thicknesses (0.5 mm, 1 mm, and 2 mm) in all implant designs: A, B, and C.

**Figure 7:**
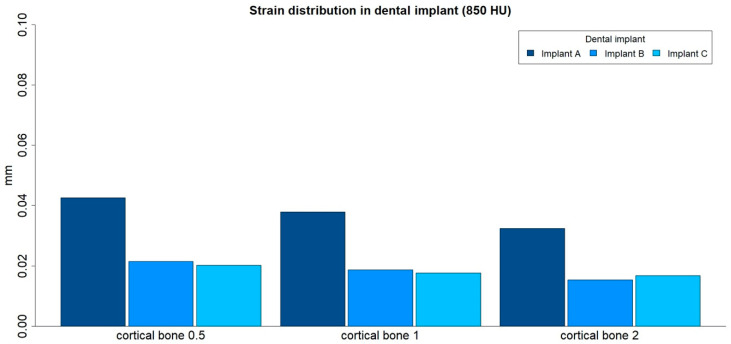
Strain distribution (mm) in implants, in high-density medullar bone (850 HU), for all cortical thicknesses (0.5 mm, 1 mm, and 2 mm) in all implant designs: A, B, and C.

The strain distributions in the implant were analyzed in both medullar bone densities (150 HU and 850 HU), for all cortical bone thicknesses (0.5 mm, 1 mm, and 2 mm), and for all implant designs (Figure 6 and Figure 7; Table 3).

The strain distribution in the dental implant was higher for implant A in all types of bone. Furthermore, the maximum principal strain in the implant decreased with an increase in cortical bone thickness for both medullar bone densities, and this difference was more prominent in the low-density medullar bone.

**Figure 8:**
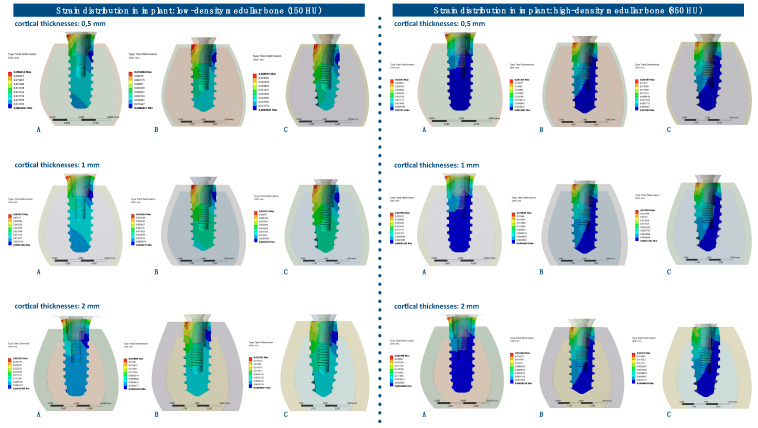
Strain distributions in implants for all implant designs: A, B, and C. Note: the figure is in very high resolution to be able to be enlarged: enlarge it in your word processor or view it in HTML format.

The distribution of the strain to the implant was concentrated, in all the examples, at the level of the most coronal portion of the implants. Likewise, there was a more homogeneous distribution of the strain throughout the body of the implant in the case of implant C (Figure 8).

### 3.3. von Mises Stress Distribution in Bone

**Figure 9:**
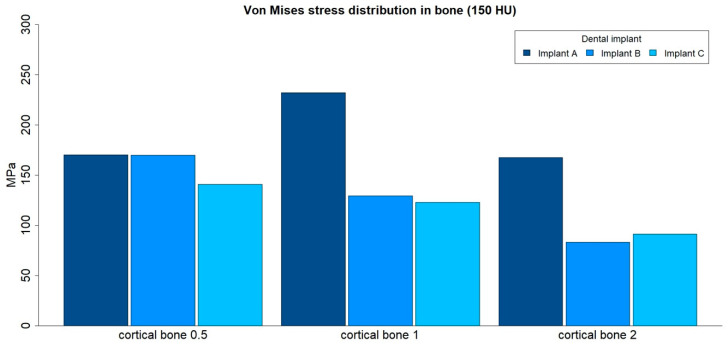
von Mises stress distribution (MPa) in low-density medullar bone (150 HU) for all cortical thicknesses (0.5 mm, 1 mm, and 2 mm) in all implant designs: A, B, and C.

**Figure 10:**
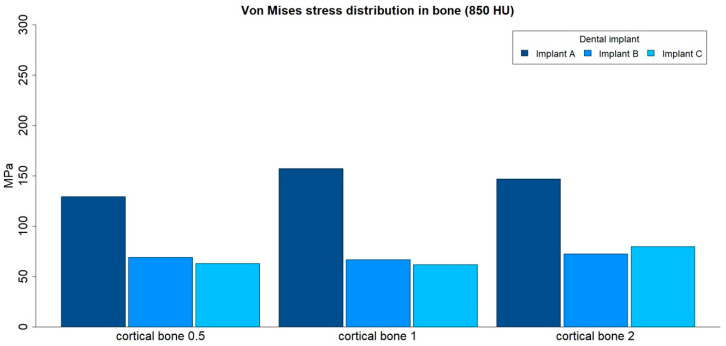
von Mises stress distribution (MPa) in high-density medullar bone (850 HU) for all cortical thicknesses (0.5 mm, 1 mm, and 2 mm) in all implant designs: A, B, and C.

The von Mises stress distributions in bone were analyzed in both medullar bone densities (150 HU and 850 HU), for all cortical bone thicknesses (0.5 mm, 1 mm, and 2 mm), and for all implant designs (Figure 9 and Figure 10; Table 4).

The von Mises stress distribution in bone was higher for implant A in all types of bone. The maximum values in stress distribution in bone decreased with an increase in medullar bone density for all implant designs.

**Figure 11:**
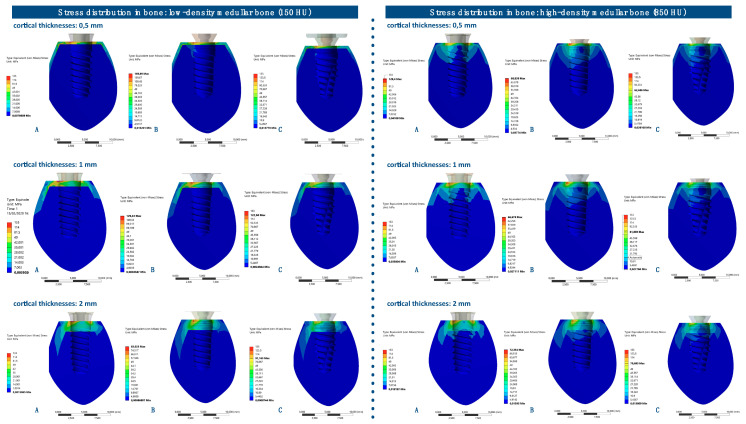
von Mises stress distributions in bone for all implant designs: A, B, and C. Note: the figure is in very high resolution to be able to be enlarged: enlarge it in your word processor or view it in HTML format.

The distribution of stress to the bone was concentrated, in all the examples, at the level of the most coronal portion of the cortical bone (Figure 11).

### 3.4. von Mises Stress Distribution in Implants

**Figure 12:**
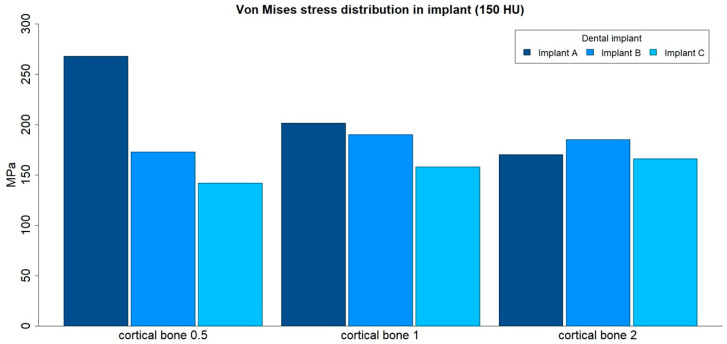
von Mises stress distributions (MPa) in implants, in low-density medullar bone (150 HU), for all cortical thicknesses (0.5 mm, 1 mm, and 2 mm) in all implant designs: A, B, and C.

**Figure 13:**
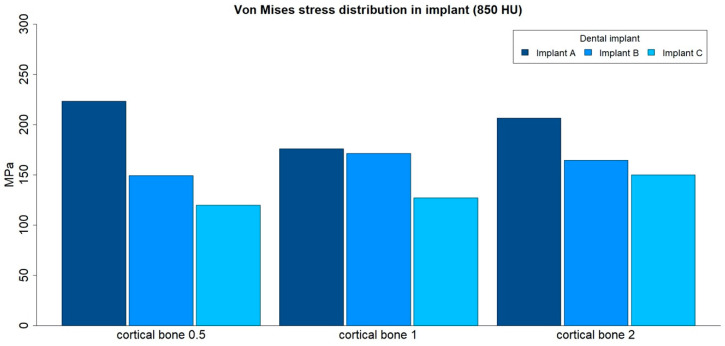
von Mises stress distributions (MPa) in implants, in high-density medullar bone (850 HU), for all cortical thicknesses (0.5 mm, 1 mm, and 2 mm) in all implant designs: A, B, and C.

The von Mises stress distributions in implants were analyzed in both medullar bone densities (150 HU and 850 HU), for all cortical bone thicknesses (0.5 mm, 1 mm, and 2 mm), and for all implant designs (Figure 12 and Figure 13; Table 5).

The von Mises stress distribution was higher for implant A in all types of bone, with the only exception for the low medullar bone density with a cortical bone of 2 mm thickness. The differences in maximum stress values between implant designs tended to be higher in low medullar bone density.

**Figure 14:**
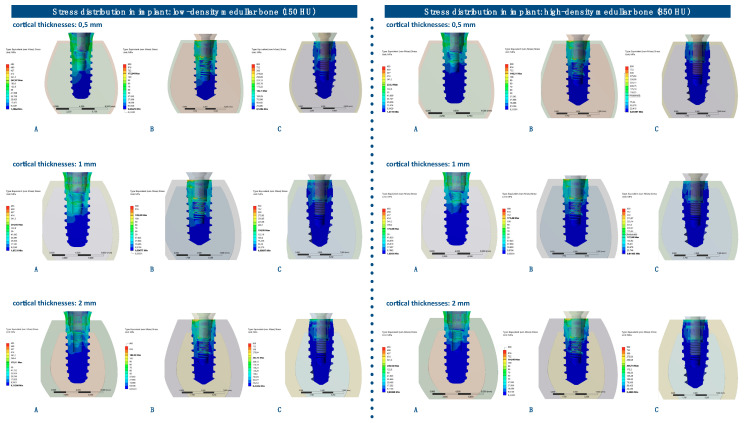
von Mises stress distributions in implants for all implant designs: A, B, and C. Note: the figure is in very high resolution to be able to be enlarged: enlarge it in your word processor or view it in HTML format.

The distribution of stress to the implant was concentrated, in all the examples, at the level of the most coronal portion of the implants. Likewise, there was a more homogeneous distribution of stress throughout the body of the implant in the case of implant C (Figure 14).

## 4. Discussion

The aim of this study was to analyze the influence of different implant designs on the stress and strain distribution to the implants and surrounding bone.

The results revealed that the strain and stress distributions in the bone and implants were noticeably affected by the implant design, medullar bone density, and thickness of the cortical bone. Therefore, the null hypothesis that all implant designs would produce similar stress and strain distributions in the implant and surrounding bone was rejected.

The maximum values of von Mises stress in bone were, in all cases, at the level of the cortical bone that surrounds the implants, being appreciably higher when the medullar bone density is lower (150 HU), and when the cortical bone is thinner (0.5 mm and 1 mm at 170.31 and 231.97 MPa, respectively). Similar data are in the study by Yalçin et al. [30], where the maximum von Mises stress value referred was on cortical bone surrounding the neck region of the implants.

A similar situation was found in the research of Araki et al. [34], with a greater stress concentration on the cortical bone around the neck of the implants, although this study was performed with shorter dental implants than those used in the present work. Herekar et al. [35] and Baggy et al. [36] also observed higher von Mises stress values located at cervical cortical bone regions adjacent to the implants for different implant types. The same results were found by Barbier et al. [37] in an animal model, showing higher stress concentrations in cortical bone at the neck of the implant, using different types of prosthetic rehabilitations.

In the present research, better results were obtained for all implant designs in high-density medullar bone (850 HU) than in low-density medullar bone (150 HU). Results for stress distribution to the bone and implants were quite similar to the strain distribution, with regard to different medullar bone densities and different cortical thicknesses. In other words, it was shown that higher densities of medullar bone and thicker cortical bone thicknesses led to lower values of stress and distribution to bone and implants, which was also reported in other studies with different implant designs and qualities of bone [28,30,36,38].

Sugiura el al. [28] showed the same pattern in their study. These authors also observed that the strain distribution was higher in low-density medullar bone and decreased with an increase in cortical thickness, in conditions of immediate and delayed loading. Works from Yalçin et al. [30] and Sevimay et al. [38] reported maximum von Mises stress values in D4 bone quality (1 mm thick cortical bone and low-density medullar bone), compared to three other bone qualities (with higher medullar densities and thicker cortical bone). Baggy et al. [36] observed higher stress distributions in the maxillary bone (less dense) than in the mandibular bone, with five different types of implants.

Other authors, however, did not report differences in stress and strain distributions in their experiments, with regards to medullar bone density. Santiago et al. [39] reported no differences in stress and strain distribution for different bone qualities (bone type III and bone type IV) in different implant designs under oblique loads. The authors suggested that this fact could be related to the thickness of the cortical bone, which was the same for the different bone qualities used in their research.

Implant design plays an important role in the success of implant treatment, helping to maintain the level of the crestal bone and facilitate the achievement of good primary stability. A correct design allows a good load distribution throughout the body of the implant. These characteristics assume greater importance in more demanding treatments such as the application of immediate loading protocols, post-extraction implants, or implants placed in low-density bones.

The implants analyzed in the present study allowed a homogeneous distribution of the occlusal loads to the surrounding bone; however, this fact was more evident for the bone-level implant designs, compared to the tissue-level implant. This may be explained by the platform-switching concept occurring in the bone-level implants used in this study, similar to the results found in the research by Maeda et al. [40], although the implants used in their work had different designs from those used in the present study. One reason that may explain this fact, according to Maeda, may be the shifting of the stress concentration away from the bone–implant interface in platform-switching concept implants [40].

Aslam et al. [41] as well as Xia et al. [42] compared a platform-matched implant with a platform-switched implant, finding a significantly higher stress distribution in peri-implant bone surrounding the platform-matched implant. A similar situation was found in this work when analyzing the behavior of implants with a tissue-level design and implants that applied a platform-switching concept to their design.

In agreement, Tabata et al. [43] showed that the von Mises stress was reduced in peri-implant bone when the platform-switching concept was applied, compared to regular platform-matched, wide platform-matched, and platform-switched concepts.

Tanasić et al. [44] also showed that the implant design without platform switching showed double the stress of the implant design with platform switching in the area of the implant neck, when compared to two different threaded implants with or without the platform-switching concept under an axial load of 500 N.

Schrotenboer et al. [45] demonstrated that when the concept of platform switching was applied by decreasing the abutment diameter, less stress was translated to the crestal bone both in microthread and smooth-neck implants under 100 N axial and oblique loads.

Contrary to the present work, Araki et al. [34] reported a higher von Mises stress distribution in bone for bone-level and tissue-level implants; however, they used other implant types and focused on extra short implants in their study.

Implant C evaluated in this study showed to be, in general, the most favorable implant design regarding stress and strain distribution to the bone and implant in most simulated situations. Furthermore, it was also observed, as a general rule, that there was a more homogeneous distribution of the stress and strain throughout the bone and the body of the implant in the case of the modified bone-level implant. This may be explained by the depth and square thread design of this specific implant, as observed in other studies [31,35].

Comparing five different implant designs under axial and oblique loads, Chun et al. [31] concluded that stress was more evenly distributed when the implant shape was square threaded and filleted with a small radius, compared to other implant designs.

Herekar et al. [35] showed that fourfold micro-threads allowed a higher stress distribution within the surrounding cortical bone and decreased stress to the medullar bone and implant body, when compared to stresses between two implants having different thread designs.

In a three-dimensional finite element analysis, Golmohammdi et al. [46] showed the importance of different lengths of the micro-threaded neck of the implants on stress distribution to the bone under an axial and oblique load (30°) of 200 N.

A previous work published by Tada et al. [47] also showed the importance of implant design and bone quality on stress and strain distribution in bone around implants. The authors suggested that screw-type implants could have a favorable response compared to cylinder implants, mainly in low-density bone. These results may lead us to think about the importance of different implant designs and their adaptation to each specific clinical situation for success in implant therapy.

This study has the limitations of being a finite element simulation. In our study, specific situations are considered (bone density, cortical thicknesses, non-axial forces, osseointegrated implants) that do not exactly reflect clinical situations. Thus, the values obtained may not correspond to the clinical behavior of the different implant designs. This type of study helps to understand the influence of implant designs on the transmission of loads to both bone and implants. It is necessary to contrast these results with those obtained in in vitro and in vivo studies. the results obtained in this study are in agreement with other similar works, which try to understand the biomechanical behavior of implants [28,30,35,38].

## 5. Conclusions

Based on the findings of this FEA study, and within the limitations of the same, one may conclude that implant design seems to affect the distribution of strain and stress in bone and implants. There was a more homogeneous strain distribution throughout the body of the implant in the case of implant C. Implant C presented lower bone strain in the cortical bone region. The density of the medullar bone, as well as the thickness of the cortical bone, also seem to affect the distribution of strain and stress in bone and implants. A decrease in medullar bone density negatively affects the strain and stress produced by the implants. All implants showed a good distribution of forces for non-axial loads with higher forces concentrated at the crestal region of the bone–implant interface. In all cases, the load distribution may be compatible with the resistance of the maxillary bone.

## Figures and Tables

**Table 1:** Material properties used in finite element models.

Implant Design	Material Component	Material	E * (GPa) #	Poisson Ratio	References
A	Implant Essential Cone^®^	Titanium Grade 3	103.4	0.340	[32,33]
B	Implant Vega^®^	Titanium Grade 4	104.0	0.340	[32]
C	Implant Vega +^®^	Titanium Grade 4	104.0	0.340	[32]
	Medullar bone (150 HU)		0.259	0.300	[28]
	Medullar bone (850 HU)		3.507	0.300	[28]
	Cortical bone		13.980	0.300	[28]

* Young’s Modulus. # Gigapascals.

**Table 2:** Strain distribution (maximum absolute values, mm) in bone for both medullar bone densities and for all cortical thicknesses (0.5 mm, 1 mm, and 2 mm) in all implant types: A, B, and C.

Bone Density	Implant Design	Cortical Bone 0.5	Cortical Bone 1	Cortical Bone 2
150 HU	A	0.061	0.038	0.019
150 HU	B	0.052	0.031	0.015
150 HU	C	0.046	0.028	0.014
850 HU	A	0.022	0.018	0.014
850 HU	B	0.018	0.014	0.011
850 HU	C	0.016	0.013	0.010

**Table 3:** Strain distribution (maximum absolute values, mm) in implants, for both medullar bone densities, for all cortical thicknesses (0.5 mm, 1 mm, and 2 mm) in all implant types: A, B, and C.

Bone Density	Implant Design	Cortical Bone 0.5	Cortical Bone 1	Cortical Bone 2
150 HU	A	0.090	0.062	0.039
150 HU	B	0.054	0.036	0.020
150 HU	C	0.049	0.033	0.021
850 HU	A	0.043	0.038	0.032
850 HU	B	0.021	0.019	0.015
850 HU	C	0.020	0.018	0.017

**Table 4:** von Mises stress distribution (maximum absolute values, MPa) in bone, for both medullar bone densities, for all cortical thicknesses (0.5 mm, 1 mm, and 2 mm) in all implant designs: A, B, and C.

Bone Density	Implant Design	Cortical Bone 0.5	Cortical Bone 1	Cortical Bone 2
150 HU	A	170.31	231.97	167.5
150 HU	B	169.89	129.43	83.03
150 HU	C	140.87	122.84	91.165
850 HU	A	129.4	157.04	146.78
850 HU	B	68.838	66.678	72.354
850 HU	C	62.686	61.803	79.693

**Table 5:** von Mises stress distributions (maximum absolute values, MPa) in implants, for both medullar bone densities, for all cortical thicknesses (0.5 mm, 1 mm, and 2 mm) in all implant designs: A, B, and C.

Bone Density	Implant Design	Cortical Bone 0.5	Cortical Bone 1	Cortical Bone 2
150 HU	A	267.87	201.66	170.11
150 HU	B	172.94	189.88	184.95
150 HU	C	142.1	158.05	166.05
850 HU	A	223.2	175.88	206.56
850 HU	B	149.21	171.46	164.48
850 HU	C	119.84	127.08	150.02
